# Tuberculin skin test surveys and the Annual Risk of Tuberculous Infection in school children in Northern KwaZulu-Natal

**DOI:** 10.1371/journal.pgph.0003263

**Published:** 2024-06-18

**Authors:** Tom A. Yates, Siphiwe Cebekhulu, Mumsy Mthethwa, P. Bernard Fourie, Marie-Louise Newell, Ibrahim Abubakar, Frank Tanser

**Affiliations:** 1 Africa Health Research Institute (AHRI), Congella, South Africa; 2 Institute of Health Informatics, University College London, London, United Kingdom; 3 Division of Infection and Immunity, University College London, London, United Kingdom; 4 Department of Medical Microbiology, University of Pretoria, Pretoria, South Africa; 5 School of Human Development and Health University of Southampton, Southampton, United Kingdom; 6 Faculty of Population Health Sciences, University College London, London, United Kingdom; 7 MRC Clinical Trials Unit, University College London, London, United Kingdom; 8 Centre for Epidemic Response and Innovation (CERI), Stellenbosch University, Stellenbosch, South Africa; 9 School of Nursing and Public Health, University of KwaZulu-Natal, Durban, South Africa; 10 Centre for the AIDS Programme of Research in South Africa (CAPRISA), University of Kwa-Zulu Natal, Congella, South Africa; JIPMER PSM: Jawaharlal Institute of Post Graduate Medical Education and Research Department of Preventive and Social Medicine, INDIA

## Abstract

Tuberculin skin test surveys in primary school children can be used to quantify *Mycobacterium tuberculosis* transmission at community level. KwaZulu-Natal province, South Africa, is home to 11.5 million people and suffers a burden of tuberculosis disease that is among the highest in the world. The last tuberculin survey in the province was undertaken in 1979. We performed a tuberculin skin test survey nested within a demographic and health household surveillance programme in Northern KwaZulu-Natal. We enrolled children aged between six and eight years of age attending primary schools in this community. Mixture analysis was used to determine tuberculin skin test thresholds and the Annual Risk of Tuberculous Infection derived from age at testing and infection prevalence. The Community Infection Ratio, a measure of the relative importance of within-household and community transmission, was calculated from data on tuberculin positivity disaggregated by household tuberculosis contact. Between June and December 2013, we obtained tuberculin skin test results on 1240 children. Mixture analysis proved unstable, suggesting two potential thresholds for test positivity. Using a threshold of ≥10mm or treating all non zero reactions as positive yielded estimates of the Annual Risk of Tuberculous Infection of 1.7% (1.4–2.1%) or 2.4% (2.0–3.0%). Using the same thresholds and including children reported to be receiving TB treatment as cases, resulted in estimates of 2.0% (1.6–2.5%) or 2.7% (2.2–3.3%). The Community Infection Ratio was 0.58 (0.33–1.01). The force of infection in this community is lower than that observed in Western Cape province, South Africa, but higher than that observed in community settings in most other parts of the world. Children in this community are commonly infected with *Mycobacterium tuberculosis* outside the home. Interventions to interrupt transmission are urgently needed.

## Introduction

High levels of *Mycobacterium tuberculosis* (*Mtb*) transmission are a major barrier to reducing the incidence of tuberculosis disease (TB) in Southern Africa [1–3]. The Annual Risk of Tuberculous Infection (ARTI), a key measure of *Mtb* transmission, is usually calculated from the prevalence of tuberculin skin test (TST) positivity in primary school aged children [4, 5]. This age group is chosen because positive tests must be the result of recent infection, because the BCG effect has usually waned in children vaccinated at birth [6, 7], and because school based testing is logistically convenient.

Here, we present data from a tuberculin school survey undertaken in 2013 in Northern KwaZulu-Natal (KZN). We explore the sensitivity of the ARTI estimate to methodological choices made. We compare our results to the last tuberculin school surveys to be undertaken in KZN, which were conducted in 1974 and 1979 [8–10]. Our objective was to quantify the force of infection in this community. We hope that these data lead to better a understanding of TB epidemiology; inform mathematical models seeking to predict the effect of interventions; and, ultimately, strengthen TB prevention in this and similar settings.

## Methods

### Setting

This study was undertaken within a large health and demographic surveillance programme in uMkhanyakude District, Northern KZN [11–14]. At the time of this survey, the population of the surveillance area was approximately 90,000 people, two thirds of whom were resident, with approximately 11,000 households.

The study area includes rural communities (approx. 20 residents per km^2^) as well as peri-urban communities close to the N2 national road (approx. 3000 residents per km^2^) [11]. In the 2011 census, uMkhanyakude was among the five most deprived districts in South Africa [15]. The main sources of income in the community are state pensions and waged employment [11]. In 2013, 72.7% of households registered in the surveillance programme drank piped water or water from a borehole and 81.1% of households were on the electrical grid.

The burden of TB disease in the community is high. In uMkhanyakude District, there were 878 notified cases of TB per 100,000 population in 2013 [16], likely an underestimate of the true burden of disease [17]. A 2018–19 TB prevalence survey estimated the prevalence of culture positive pulmonary TB in this community to be 562 per 100,000 population [18]. TB is a leading cause of death in the community [19]. In 2011, 29% of adults registered in the surveillance programme were HIV positive [13, 20].

### Population

To be eligible for TST testing, children had to be born in 2005 or 2006 (i.e. between six and eight years old at the time of the survey), registered in the Africa Health Research Institute (AHRI) surveillance programme and, to minimise disruption in schools, attending Grade 1 or 2 in one of the thirty-eight primary or lower primary schools in the surveillance area. Where children in Reception or Grade 3 were erroneously consented, we tested them as per their parent or guardian’s wishes. Children reported to currently be receiving TB treatment were not eligible. For the purposes of this analysis, we have only included children who were resident in the surveillance area on 25 June 2013, the day we began consenting children for this study.

### Study recruitment

Written consent for testing was obtained from a parent or caregiver in one of two ways. First, we attempted to consent children during a routine visit to the household by an AHRI fieldworker. Where this was not possible, letters about the study and consent forms, in English and isiZulu, were sent home from schools with children. At the request of the ethics committee, we also obtained written assent from each child.

The identities of children were confirmed prior to testing by asking them to confirm three or more of the following—child’s name, child’s date of birth, mother’s name, the name of the head of household, the area in which their homestead was located, or the Bounded Structure ID. The Bounded Structure ID is a unique identifier used by AHRI to identify homesteads. A piece of plastic with this number on it is usually tacked onto the door or gatepost.

### Tuberculin testing

We administered 2 tuberculin units of RT23/Tween 80 (Statens Serum Institut, Copenhagen, Denmark) into the volar aspect of the forearm of each child with a 26 gauge needle. Skin was cleaned with alcohol only if visibly dirty. Tests were not administered if children had a febrile illness or skin disease.

We aimed to read tests at 72 hours, though permitted tests to be read between 48 and 96 hours. Measurement of the maximum transverse diameter of any induration was made, to the nearest millimetre, with a transparent ruler. After measuring the TST reaction, the study nurse then inspected for a BCG scar.

### Clinical management of positive reactions

In South Africa, immunocompetent children over the age of five are not treated for latent tuberculosis infection. Children in our survey with TST reactions of ≥10mm, or ≥5mm in HIV positive children, were advised to attend the TB clinic at the district hospital for assessment if there was a history of recent TB contact, if the child was HIV positive, if they had symptoms or signs of TB, or if they were failing to thrive. Participants could use a Department of Health bus service to travel to the hospital, and the study paid travel costs for the return journey. There was a dedicated study phone that participants’ families could use to contact the research team.

### Statistical analysis

Using data collected by the surveillance programme, we described differences between children eligible for enrolment into the study for whom we had or did not have a TST result. Our approach to defining urban versus rural residence and household wealth is detailed below. In describing these differences, household refers to the homestead in which the child was living on 25 June 2013, the day we started enrolment.

Small TST reactions can be seen following exposure to non-tuberculous mycobacteria (NTM) or to BCG vaccine. In populations where both *Mtb* infections and these ‘non-specific’ reactions are seen, the distribution of non zero TST reaction sizes tends to be bimodal. Overlap between the two distributions is seen, therefore some misclassification is inevitable [4, 21]. However, choosing an appropriate cut point will result in estimates of *Mtb* infection prevalence that are approximately correct, balancing ‘errors resulting from a lack of sensitivity against errors from a lack of specificity’ [4].

Our primary measure of TST positivity used cut points determined using mixture analysis [22–24]. The approach is attractive as it allows an analysis plan to be pre-specified where little is known about the prevalence of *Mtb* infection and non-specific reactions in the population of interest. Tuberculin reactions in people exposed to *Mtb* are approximately normally distributed [25]. It has been argued that non-specific reactions in people exposed to environmental mycobacteria are also approximately normally distributed [24]. Here, we used the *normalmixEM* tool in R (R Foundation for Statistical Computing, Vienna, Austria) to fit a two component normal model to the observed distribution of non zero reactions [26]. In the main model, we only placed constraints on the number of components and that the means of these two distributions should differ and both be of positive sign.

Given the inherent difficulties in determining TST cut points [4, 21, 24, 27], we also present ARTI estimates using several alternative approaches advocated in the literature [21] as per Shanaube *et al*. [28]. A brief description of each of these approaches can be found in S1 Text.

Using each threshold, estimates of infection prevalence and their associated 95% confidence intervals were calculated in STATA version 14.2 (Stata Corp, College Station, Texas, USA). We accounted for clustering by school using robust standard errors.

For each of the ‘mirror methods’ [21, 28], we used the same approach for reactions equal to or greater than the mode. We then repeated this for reactions greater than the mode. Conservative confidence intervals were then obtained by summing the ends of the two sets of 95% confidence intervals.

We planned to present prevalence estimates stratified by age in years, sex, urban versus rural residence, quantiles of household asset ownership, by whether the child had ever lived outside the surveillance area, and by whether the child was reported to have had previous household contact with someone with TB. Urban areas are formally designated as such, with periurban residence defined as living in a non urban area with a population density of ≥400 residents per km^2^.

A household asset index was calculated by principal components analysis (PCA) [29], using 2013 data on each homestead. A list of assets included in the PCA and a brief critique of this approach to measuring wealth in this population can be found in S1 Text. The PCA included data on every household in the surveillance area, not just those in which children in our study were resident. We opted against using maternal or head of household educational attainment as our measure of socioeconomic position as many households in this community are headed by a grandparent, and educational opportunities for black people in South Africa changed substantially after 1994.

Confidence intervals for TST prevalence within subgroups were calculated using the same approach as for the sample as a whole, using robust standard errors to account for clustering by school. This approach considered the correlations within the whole dataset, not just between children in each stratum.

ARTI was calculated as per Nyboe [30] using

$$ARTI=1-{\left(1-prevalence\right)}^{\wedge }(1/mean\;age\;at\;testing)$$

This assumes that force of infection does not vary by age and that it has been constant over the period that these children have been alive.

We generated confidence intervals for age at testing, using robust standard errors to account for clustering by school. Conservatively, confidence intervals for the ARTI estimate were generated by taking values from the ends of the 95% confidence intervals for both prevalence and mean age at testing.

Children reported to be receiving TB treatment were not tested. Separately, we generated a second set of infection prevalence and ARTI estimates with these children included, under the assumption that their TST reactions would have been positive. In calculating the ARTI, children reported to be receiving TB treatment were assigned the age they had on the date they were reported to be receiving TB treatment.

Using the approach described by Madico [31], we calculated the Community Infection Ratio (CIR), ‘an index of the relative importance of within-household and community spread of infection.’ Mathematically, this is an odds ratio comparing the odds of infection in individuals with no history of household contact to that in individuals who report household exposure to TB.


$$CIR=\frac{\left(prevalence\;in\;controls\;)/(1-prevalence\;in\;controls\right)}{\left(prevalence\;in\;contacts)/(1-prevalence\;in\;contacts\right)}$$

We obtained conservative confidence intervals for the CIR by taking the ends of the confidence intervals for prevalence in both controls and contacts that both maximised and minimised the CIR estimate. We did not obtain data on TB contact from children not eligible for enrolment into our TST survey, which included children reported to be currently in receipt of TB treatment. We therefore undertook a sensitivity analysis calculating the CIR under the assumption that either all of these children or none had a history of household TB contact.

Univariable odds ratios describing associations between key child and household characteristics and TST positivity were calculated using mixed effects logistic regression models that accounted for clustering at both household and school levels. Here, household refers to the homestead within the study area in which the child lived for the longest period of time. As there was very little missing data on these child and household characteristics, we opted to present a complete case analysis.

### Historical comparisons

To our knowledge, the most recent previous tuberculin school surveys undertaken in KZN were in 1974 and 1979 [8–10]. We were able to access summary level results from the authors. We present data from these surveys for 5–9 year old black school children living in rural KZN. These children were recruited from rural parts of Tugela Ferry, Ulundi, Nongoma, Empangeni, Hlabisa, Jozini, Mkuze and Hluhluwe–all communities in Northern KZN. There were few 5 and 6 year olds attending school, meaning the median age was 7.5 to 8 years.

The data from the 1974 and 1979 surveys were provided aggregated into 2mm bins, with non reactions included in the lowest category (0–1.9mm). To format the 2013 data in the same way necessitated partitioning some of the observations–e.g. half of the 2mm reactions were included in the 0–1.9mm category with the other half included in the 2–3.9mm category.

### Ethics

Approvals to undertake our 2013 tuberculin survey were obtained from the University of KwaZulu-Natal Biomedical Research Ethics Committee (BF075/13), the AHRI Community Advisory Board, the medical director at Hlabisa Hospital and the Department of Education (Hlabisa Circuit). Details of how we obtained written consent from a parent or guardian plus assent from each child are given above.

We made four protocol amendments following initial ethical approval, all concerning the logistics of fieldwork or the management of children with positive TST tests. There were two protocol breaches during data collection–a child who was erroneously tested despite being on TB treatment, and four study documents that were lost between data capture and digitisation. All protocol deviations were reported to the ethics committee.

Study authors (TAY, SC, MM) had access to data that could identify individual participants during data collection. Following data collection, the analysis was undertaken using pseudonymised data extracts.

### Data and code availability

Summary level data from the 1974 and 1979 surveys are included in S1 Text. Individual level TST data from the 2013 survey, which can be linked to other AHRI datasets, are available via the AHRI Data Repository (https://data.ahri.org). The code used for the mixture analysis is available at https://github.com/tayates/kzn_tst.

### Inclusivity in global research

Additional information regarding the ethical, cultural, and scientific considerations specific to inclusivity in global research is included in S2 Checklist.

## Results

### Survey participation

**Fig 1:**
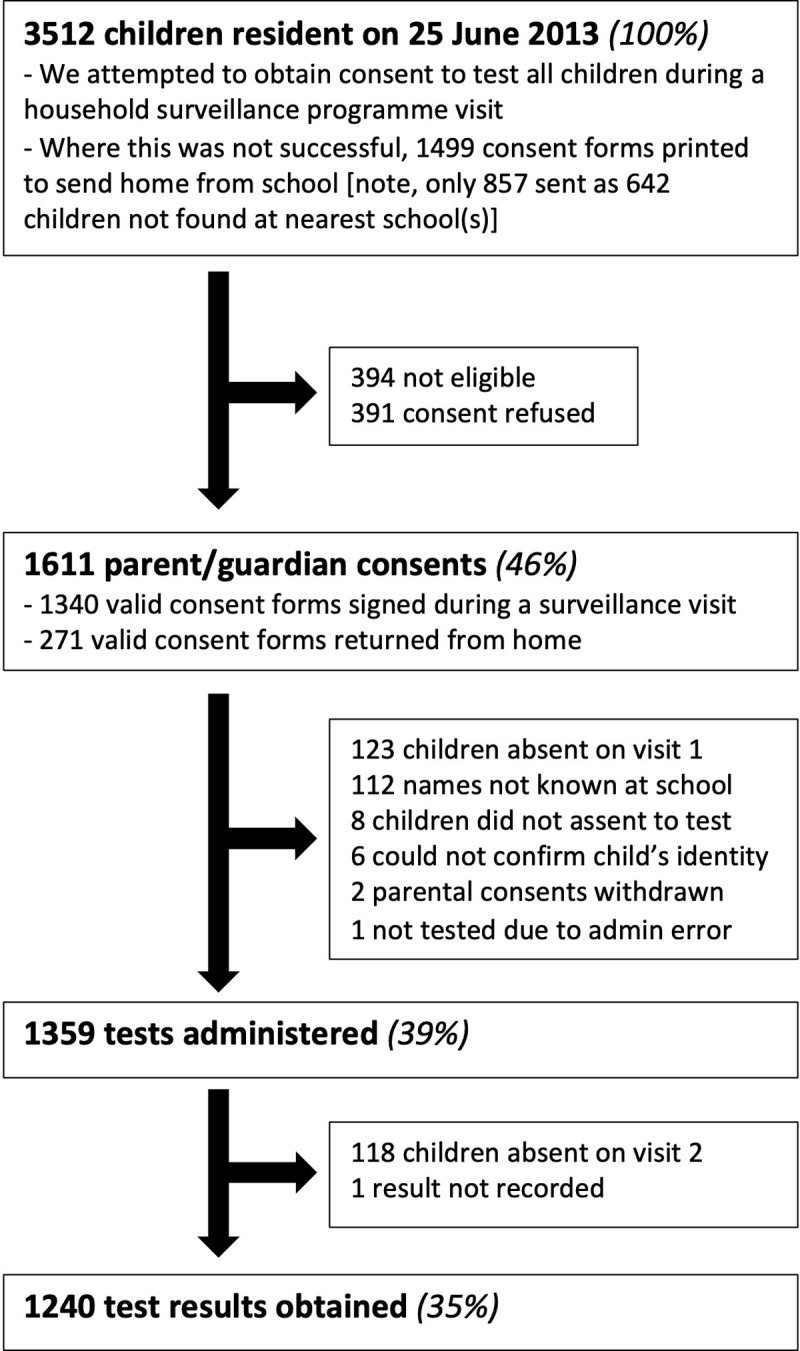
Flow chart describing survey participation. All percentages are given as a proportion of all eligible children resident in the surveillance area at the start of study enrolment.

The survey was undertaken between June and December 2013. Survey participation and losses to follow up at each stage are described in Fig 1. The commonest reason for children not being enrolled was that no parent or guardian was present when the fieldworker visited their homestead. Limited additional consents were obtained by sending letters home from school. It was common to not find record of children at the schools nearest their homes and, where letters were sent home with children, we were unable to tell whether these letters reached parents or guardians.

A comparison between children resident at the start of enrolment for whom we had a TST result and those for whom we did not is presented in Table 1. We were less likely to have TST results for children living in periurban areas as compared to children living in rural areas, slightly less likely to have results on eight year olds, and slightly less likely to have data on children living in more asset rich homesteads.

**Table 1:** The characteristics of children resident at the start of enrolment for whom we had versus did not have a TST result.

	Number of children with TST result (column %)	Number of children without a TST result (column %)	p value
Overall	1240 (100)	2272 (100)	
Sex			
Male	622 (50)	1150 (51)	0.80
Female	618 (50)	1122 (49)	
Age in years			
6	339 (27)	545 (24)	<0.0001
7	639 (52)	1047 (46)	
8	262 (21)	680 (30)	
Residence			
Urban	73 (6)	171 (8)	<0.0001
Peri-urban	290 (24)	814 (36)	
Rural	868 (71)	1260 (56)	
Household wealth			
Poorest quintile	313 (26)	357 (17)	<0.0001
Second quintile	229 (19)	423 (20)	
Third quintile	248 (21)	445 (21)	
Fourth quintile	245 (21)	464 (22)	
Wealthiest quintile	159 (13)	418 (20)	
Residence outside surveillance area			
Never	968 (78)	1767 (78)	0.84
Ever	272 (22)	505 (22)	
BCG vaccine			
Vaccinated	1022 (100)	1762 (100)	0.15
Not vaccinated	1 (0)	8 (0)	

Here, age and residence are based on data from 25 June 2013, the day we began to consent children. The p values are Wald tests from a logistic regression model. Note, there is some missing data on residence (1%), household wealth (6%), and documented receipt of BCG vaccine (20%).

The 38 schools varied considerably in size. At the smallest rural school, we successfully read only one TST whereas, at the largest schools close to the national road, we read 114 and 166 TSTs.

### Distribution of TST results and inspection for BCG scars

**Fig 2:**
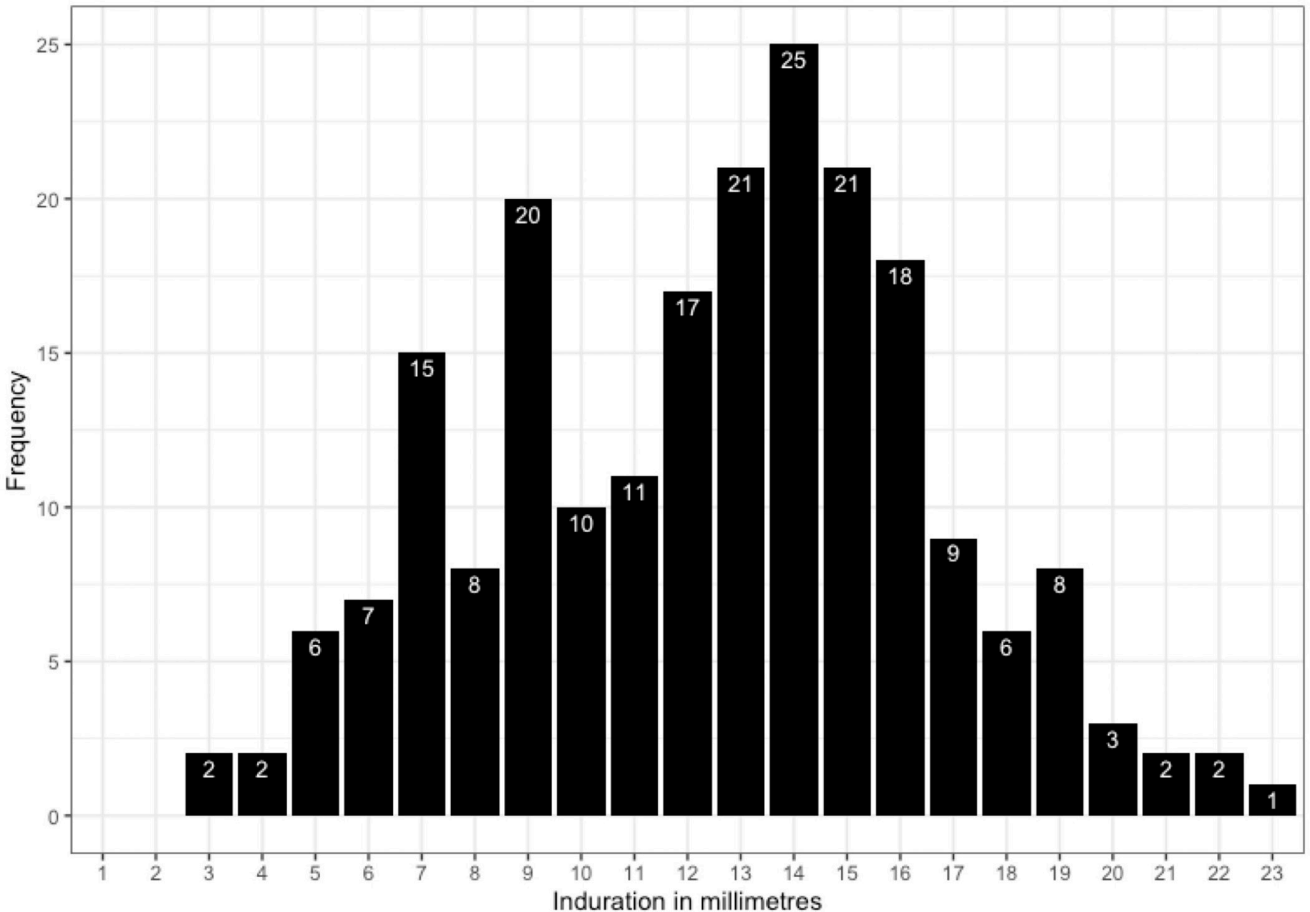
The distribution of TST results in the 214 children from the 2013 survey with non-zero reactions.

Of the 1240 children for whom we had TST results, 214 had non-zero reactions (Fig 2). There were a small number of 3-4mm reactions and the largest reaction observed was 23mm. The mode of the distribution was at 14mm. There was a suggestion that there might be inverse digit preference–i.e. readers seeking to avoid the 10mm and 15mm readings that, historically, have been favoured–with peaks seen at 9mm and 14mm. However, with small numbers in each bracket, this pattern could have occurred by chance.

Of these 1240 children, 1119 had a BCG scar visible (90.2%), a further 73 (5.9%) had a doubtful BCG scar, and 47 (3.8%) had no visible BCG scar (one observation was missing). Due to small numbers, we elected not to present data on the children without BCG scars separately.

### Mixture analysis

**Fig 3:**
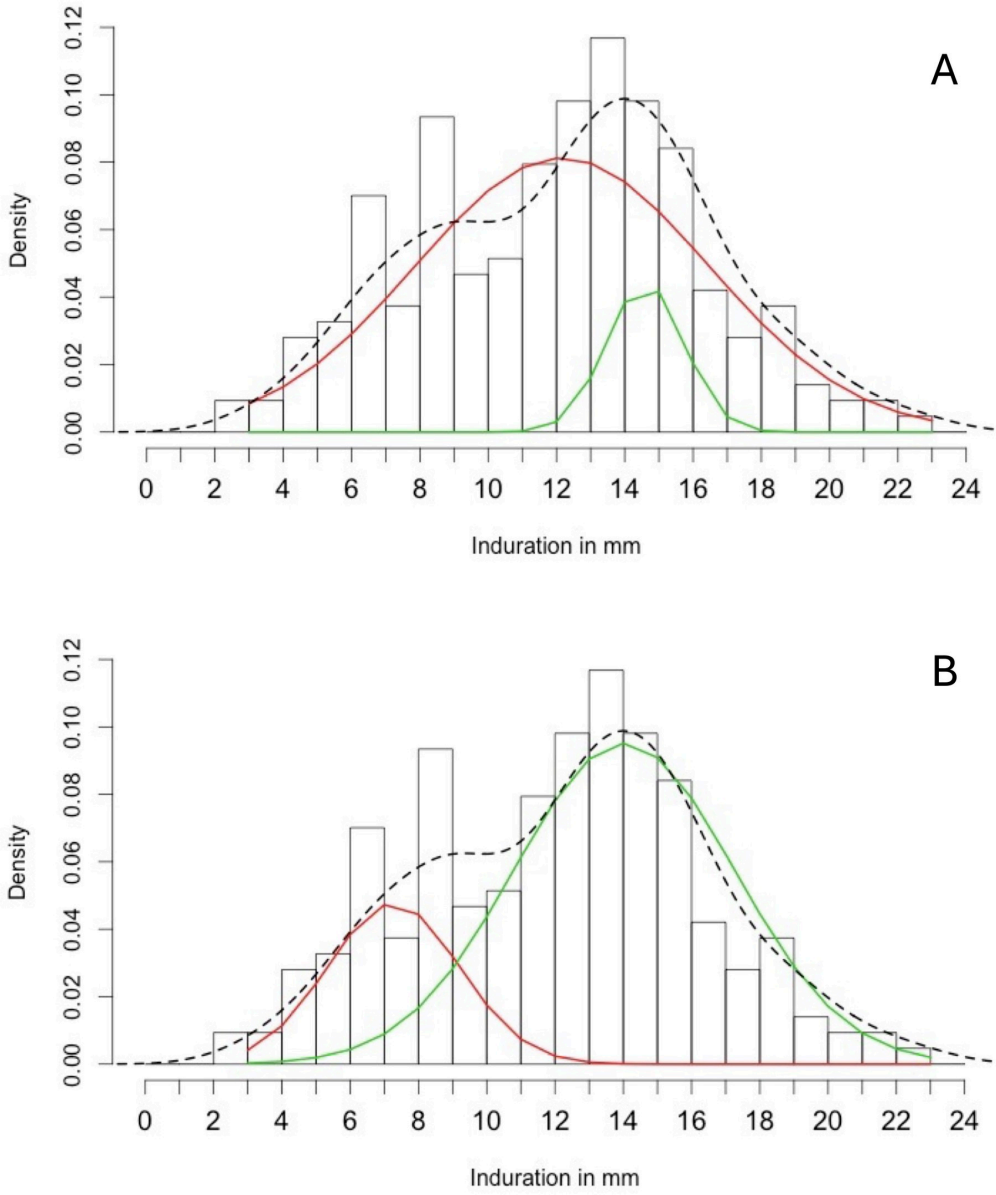
Two sets of results from the same mixture analysis model, both using data on the 214 children in the 2013 TST survey with non-zero reactions. The dashed line is a smoothed representation of the data. The red and green lines are component normal distributions. Model output proved unstable, yielding two potential sets of results, (A) and (B).

The results of the mixture analysis were unstable. This was because the ‘seed’ used to initialise parameter estimation was not specified. The same code yielded one of two sets of component distributions depending on the seed randomly selected by the *normalmixEM* function (Fig 3).

The overlapping distributions of the two components in the upper panel is consistent with the data coming from a single distribution. This result would suggest that all non-zero TST readings might be considered to represent *Mtb* infection. The alternative result suggests a smaller component with a mode at 7mm with the majority of positive reactions falling within a larger component with a mode at 14mm. This result would suggest some non-specific reactions and that a threshold of ≥10mm, which is where the curves intersect, would give an estimate of infection prevalence that is approximately correct [4].

In response to reviewer comments, we explored whether specifying starting values of vector of component means might resolve difficulties in identifying the underlying distributions. Constraining the model to have two component distributions, the output remained unstable with the starting values of the smaller component at 3mm, 5mm or 7mm, and with the starting values of the larger component at 14mm, 16mm or 18mm, including all permutations. We explored whether smoothing the data to account for potential digit preference using a three point rolling average [4] might improve model instability–it did not. We also ran a three component normal model, e.g. BCG, NTM and *Mtb*. This model was stable but the results, suggesting two of the three discrete component distributions had means considerably greater than 10mm, made no biological sense in a population of children that had not recently received BCG vaccine [6, 7, 22].

### Infection prevalence, ARTI and the CIR

Estimates of infection prevalence using both thresholds suggested by mixture analysis are presented in Table 2. These are presented with and without including children reported to be receiving TB treatment in the sample. Also presented are infection prevalence estimates using a set of thresholds taken from the published literature [21, 28].

**Table 2:** Estimates of infection prevalence using various approaches to define test positivity.

	All positive reactions	10mm	14mm x 1.22	15mm	Mirror (mode 14mm)	Fixed mirror (at 17mm)	All positive reactions plus those on TB treatment	10mm plus those on TB treatment
**Overall**	17.3 (14.3–20.7)	12.4 (10.2–15.0)	9.3 (7.2–12.0)	5.6 (4.1–7.7)	13.3 (10.1–17.5)	4.3 (2.6–7.1)	19.1 (15.8–22.9)	14.4 (11.8–17.4)
**Sex**								
Male	15.8 (11.8–20.7)	11.4 (8.3–15.4)	9.0 (6.2–13.0)	5.5 (3.6–8.3)	12.9 (8.6–19.0)	4.5 (2.4–8.6)	18.4 (13.9–23.8)	14.2 (10.5–18.8)
Female	18.8 (15.6–22.5)	13.4 (10.9–16.4)	9.7 (7.1–13.1)	5.8 (3.8–8.7)	13.8 (9.7–19.5)	4.0 (2.1–7.7)	19.8 (16.2–24.0)	14.5 (11.6–18.0)
**Age in years**								
6	18.1 (12.3–25.7)	11.8 (7.3–18.6)	5.9 (2.7–12.7)	4.9 (2.2–10.4)	9.7 (4.4–20.9)	2.8 (0.7–11.2)	22.4 (16.7–29.3)	16.4 (11.4–23.2)
7	16.7 (13.5–20.6)	12.6 (10.0–15.6)	11.2 (8.8–14.2)	6.9 (5.2–9.3)	16.1 (12.4–20.9)	5.3 (3.3–8.6)	18.2 (14.6–22.3)	14.1 (11.2–17.6)
8	17.9 (12.9–24.2)	12.4 (8.9–17.0)	7.6 (4.5–12.4)	3.8 (2.0–7.0)	10.0 (5.7–17.2)	3.1 (1.3–7.4)	19.4 (14.2–26.0)	14.0 (10.2–18.9)
**Residence (2013)**								
Urban	28.8 (23.6–34.5)	19.2 (13.6–26.3)	15.0 (7.6–28.1)	9.6 (5.1–17.4)	21.9 (11.3–40.4)	9.6 (4.6–19.5)	30.7 (24.6–37.4)	21.3 (14.8–29.8)
Peri-urban	17.2 (11.6–24.8)	14.1 (9.9–19.8)	11.4 (8.1–15.8)	7.2 (4.3–12.0)	16.6 (10.9–24.9)	4.8 (2.5–10.1)	18.1 (11.8–26.7)	15.0 (10.1–21.8)
Rural	16.4 (13.8–19.3)	11.3 (9.3–13.7)	8.2 (6.1–10.8)	4.7 (3.3–6.8)	11.4 (8.2–15.6)	3.5 (1.8–6.7)	18.5 (15.7–21.7)	13.6 (11.1–16.5)
**Household wealth (2013)**								
Poorest quintile	17.6 (13.1–23.1)	12.1 (8.8–16.6)	9.4 (5.9–14.7)	5.4 (3.1–9.3)	13.1 (7.9–21.4)	4.8 (1.7–13.2)	19.9 (15.5–25.1)	14.6 (11.3–18.7)
Second quintile	14.8 (11.3–19.3)	12.2 (9.3–16.0)	9.6 (6.8–13.3)	4.8 (2.9–7.8)	12.7 (8.6–18.7)	3.9 (1.7–9.2)	16.3 (12.6–20.9)	13.7 (10.5–17.8)
Third quintile	21.4 (16.2–27.7)	16.1 (11.9–21.6)	10.8 (6.8–16.9)	5.6 (3.0–10.3)	14.5 (8.6–24.1)	5.2 (2.2–12.5)	22.9 (17.2–29.9)	17.8 (13.1–23.7)
Fourth quintile	17.1 (12.1–23.7)	10.6 (6.8–16.2)	8.0 (4.2–14.6)	5.3 (2.7–10.2)	11.8 (6.2–22.2)	2.9 (1.0–7.7)	18.1 (12.9-24-9)	11.7 (7.6–17.5)
Wealthiest quintile	16.4 (10.8–23.9)	11.9 (8.2–17.1)	10.0 (6.6–14.9)	8.2 (5.4–12.2)	16.4 (10.8–24.5)	5.7 (2.6–12.4)	17.9 (12.5–25.0)	13.6 (9.8–18.5)
**Residence outside surveillance area**								
Never	16.3 (13.2–20.0)	11.9 (9.7–14.5)	9.1 (6.9–11.8)	5.7 (4.0–8.0)	13.1 (9.7–17.7)	4.1 (2.4–7.2)	18.1 (14.5–22.3)	13.8 (11.0–17.0)
Ever	20.6 (15.7–26.6)	14.3 (10.9–18.6)	10.3 (6.9–15.3)	5.5 (3.2–9.2)	14.0 (8.9–21.7)	4.8 (2.1–10.9)	22.6 (17.8–28.2)	16.5 (13.1–20.5)
**History of household TB contact**								
No	15.4 (12.4–19.0)	11.1 (8.7–14.0)	7.9 (5.9–10.6)	4.7 (3.2–6.8)	11.1 (8.0–15.5)	3.8 (2.4–6.2)	Not calculated	Not calculated
Yes	23.9 (18.9–29.8)	17.5 (13.4–22.5)	14.4 (10.6–19.3)	8.9 (6.1–12.9)	20.7 (14.8–28.7)	6.1 (2.5–14.3)	Not calculated	Not calculated

All estimates adjusted for clustering by school using robust standard errors. Here age is age at testing except for calculations including children reported to be on TB treatment, where I used age on 25 June 2013. Note, history of TB contact was only obtained after children consented to participate in tuberculin survey, and current receipt of TB treatment made children ineligible to be enrolled into the survey.

Using the thresholds suggested by mixture analysis, the ARTI was estimated to be 1.7% (1.4–2.1%) or 2.4% (2.0–3.0%). Using the same thresholds and including 28 children reported to be receiving TB treatment as cases, the ARTI was estimated to be 2.0% (1.6–2.5%) or 2.7% (2.2–3.3%).

Using the thresholds suggested by mixture analysis, the CIR was 0.59 (0.33–1.05) or 0.58 (0.33–1.01). In sensitivity analysis, assuming all children reported to be on TB treatment had *Mtb* infection but no history of household TB contact yielded a CIR estimates of 0.74 (0.41–1.35) or 0.69 (0.39–1.22). Repeating this analysis, assuming these children had *Mtb* infection and all had a history of household TB contact resulted in CIR estimates of 0.37 (0.21–0.65) or 0.41 (0.24–0.70).

### Participant characteristics associated with TST positivity

Selected univariable associations between key covariates and TST positivity are presented in Table 3. A full set of univariable and multivariable regression models, plus a set of spatial analyses, are presented elsewhere [32]. The multivariable and spatial analyses were underpowered and the results should be considered hypothesis generating.

**Table 3:** Univariable associations between TST positivity and key child and household characteristics.

	Infection defined as a non zero TST			Infection defined as TST ≥10mm		
	n/N (%)	OR (95% CI)	p value	n/N (%)	OR (95% CI)	p value
Age at testing in years						
6	26/144 (18)	Referent	0.93	17/144 (12)	Referent	0.25
7	113/676 (17)	0.96 (0.52–1.76)		85/676 (13)	1.33 (0.60–2.13)	
8	75/420 (18)	1.04 (0.55–1.97)		52/420 (12)	1.11 (0.57–2.15)	
Sex						
Male	98/622 (16)	Referent	0.18	71/622 (11)	Referent	0.28
Female	116/618 (19)	1.30 (0.88–1.91)		83/618 (13)	1.24 (0.84–1.82)	
Homestead size (number of residents)						
1–9	88/438 (20)	Referent	0.26	56/438 (13)	Referent	0.70
10–15	67/421 (16)	0.72 (0.46–1.14)		48/421 (11)	0.89 (0.55–1.43)	
16+	59/381 (15)	0.72 (0.45–1.14)		50/381 (13)	1.10 (0.68–1.79)	
Household wealth score (quintiles)						
1 (poorest)	45/260 (17)	Referent	0.19	28/260 (11)	Referent	0.19
2	44/293 (15)	0.80 (0.44–1.46)		32/293 (11)	1.00 (0.55–1.83)	
3	57/258 (22)	1.55 (0.85–2.85)		44/258 (17)	1.84 (1.00–3.37)	
4	36/242 (15)	0.87 (0.46–1.63)		28/242 (12)	1.14 (0.60–2.14)	
5 (richest)	31/179 (17)	1.14 (0.58–2.27)		22/179 (12)	1.26 (0.63–2.50)	
Residence outside surveillance area						
Never	158/968 (16)	Referent	0.14	115/968 (12)	Referent	0.34
Ever	56/272 (21)	1.39 (0.89–2.17)		39/272 (14)	1.24 (0.80–1.94)	
History of TB contact in the household						
No	145/941 (15)	Referent	0.002	104/941 (11)	Referent	0.007
Yes	67/280 (24)	2.04 (1.24–3.38)		49/280 (18)	1.82 (1.15–2.86)	

The odds ratios are taken from mixed effects logistic regression models accounting for clustering at both household and school level. The p values are from likelihood ratio tests. Here, age is age at testing, and the household level variables are those associated with the homestead in which the child had lived for the longest period of time. There are missing data on household wealth (1%) and history of TB contact in the household (2%).

TST positivity was associated with history of household TB contact. Higher prevalence of *Mtb* infection in older children was unexpectedly not seen. This is likely a result of the narrow age range and there being few six year olds in the sample. The apparent higher prevalence of *Mtb* infection in children resident in urban areas should be treated with caution, as there were only 73 children in this group.

### Historical comparison

**Fig 4:**
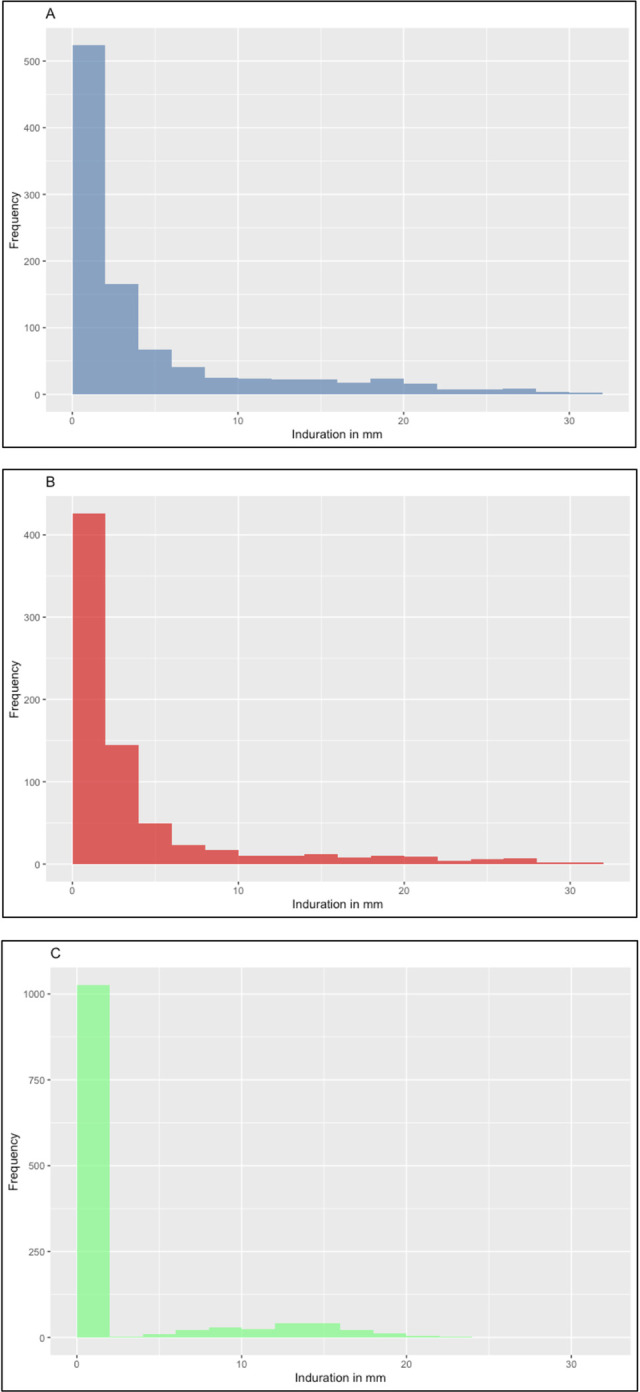
Histograms showing data from tuberculin school surveys in KZN in (A) 1974 and 1979, all children (n = 976); (B) 1974 and 1979, only children without a BCG scar (n = 741); and (C) 2013, all children (n = 1240). In all of these histograms, children with non reactive TSTs are included in the bottom category.

Fig 4 shows the reformatted 2013 data alongside data from the 1974 and 1979 surveys. Because there were limited data from 5–9 year old children in the earlier surveys, the data from 1974 (n = 523) and 1979 (n = 453) are combined here. The data are grouped into 2mm intervals, as described above, including children with non-reactions in the first bin. The data from the earlier surveys are disaggregated by whether or not a BCG scar was visible. This was not done for the 2013 survey, as there were very few children with no BCG scar. The striking differences between these distributions and the reasons we were unable to formally quantify changes in ARTI over time are discussed below.

## Discussion

We present the first estimates of the force of tuberculous infection in KwaZulu-Natal since 1979. Mixture analysis proved unstable but this had a limited effect on the estimate of ARTI, which was estimated to be 1.7% (threshold ≥10mm) or 2.4% (all positives). Including children reported to be receiving TB treatment as cases increased these estimates to 2.0 or 2.7%.

Not setting a seed for our mixture analysis proved fortuitous, as it alerted us to model instability. We would now advocate this approach. Model instability is expected where, as here, the sample size is relatively small [23]. Regards which of the two distributions is ‘correct’, we are inclined–based on prior knowledge rather than the inconsistent results of the mixture analysis–to believe that the distribution of TST reactions in KZN is unimodal. The absence of small reactions in our 2013 survey is not consistent with TST distributions seen in settings with a clear bimodal distribution of positive reaction sizes [21, 22]. Unimodal TST distributions are observed in more temperate parts of South Africa [28, 33, 34].

We did not attempt mixture analysis using other component distributions, e.g. Weibull or log-normal, but don’t have reason to believe that this would improve model stability. Use of Weibull or log-normal distributions for the non-specific reaction component has been advocated, as normal distributions can include negative values [23]–this did not seem to be an issue in our data. Code for implementing mixture analysis with Weibull or log-normal components, is available elsewhere [23].

The CIR estimate from this survey, 0.58 (0.33–1.01), was sensitive to assumptions about household TB contact in children reported to be in receipt of TB treatment. However, even under the most extreme assumptions, the CIR was no lower than 0.37 (0.21–0.65). This compares to an estimate of 0.40 (95% Cl 0.26–0.64) from Lima, Peru, and estimates of 0.18–0.37 from lower incidence settings [31]. In keeping with this, the crude odds ratio for TST positivity by history of household TB contact was closer to the 2.1 seen in surveys from settings with a TB prevalence >500 per 100,000 per annum, than to the 6.0 seen in surveys from settings where prevalence is <250 per 100,000 per annum [35]. This is consistent with a growing body of evidence that suggests, as for adults [32, 36], most children–particularly in high transmission settings–acquire *Mtb* outside the household [37]. Of note, 22.6% of the 1240 children for whom we had TST results had a history of household TB contact.

The differences in the TST distributions seen in KZN in the 1970s [8–10] as compared to 2013 are likely explained by BCG vaccination. In the 1970s, BCG was given via multiple puncture tools (Heaf guns) rather than intradermal injection, which might be expected to result in less scarring and also less sensitisation to tuberculin [38]. However, at the time, BCG was given not only at birth but also at school entry and school leaving. With the BCG effect known to wane with time since vaccination [6, 7], the preschool BCG dose might explain the disproportionate number of small TST reactions seen in the 1974 and 1979 surveys which were not observed in the 2013 survey.

These marked differences in the distributions of positive reactions meant standard methods could not be used to describe trends in ARTI over time in this community. The Rust and Thomas approach [39, 40], which may allow such comparisons [7], was not possible because the 1974 and 1979 survey data grouped zero reactions with other small (<2mm) reactions. We, therefore, opted simply to present graphical comparisons. With birth doses of BCG now given to almost all children in this community, we would expect a repeat tuberculin school survey to provide data that could be compared to our 2013 study.

Caution should be exercised in using TST surveys to make inference about between community differences in the force of infection [24, 27]. Artefactual differences may result from differences in the relative prevalence of *Mtb* and NTM infection or choices about the TST threshold used to define positivity. Furthermore, between country differences in the contact primary school age children have with other members of their community will also impact the extent to which TST positivity in this group reflects rates of transmission in the community [41, 42]. Transmission from adult men plays an important role in TB epidemiology in many communities [41, 43, 44].

That said, the ARTI estimated in our survey was clearly lower than that estimated in tuberculin surveys recruiting primary school children in the Western Cape of South Africa. There, three recent surveys have estimated an ARTI of between 3.7% and 4.2% in children of a comparable age (unimodal distributions, ≥10mm threshold)–these surveys were all undertaken in and around Cape Town [28, 33, 34]. The ARTI we estimated was similar to that estimated in a recent survey in Zambia, where a 2.0% ARTI was calculated using the same threshold (bimodal distribution) [28]; to the 2.2% ARTI estimated in Somalia, using a model to address both non-specific reactions and digit preference (bimodal distribution) [45]; and to the 2.6–3.1% ARTI estimates obtained for Djibouti using various approaches (inexplicably, the distribution of positive reactions in Djibouti appeared very different in 2001 to that seen in 1994) [46]. The ARTI we estimated in KZN seemed higher than the 0.7% estimated in Tanzania and the 1.1% estimated in Kenya (bimodal distributions, both surveys used the fixed mirror method) [47, 48], the 0.8% estimated in Gambia (analysed as a bimodal distribution, ≥12mm from mixture analysis) [49], ARTI estimates from a survey in Ghana, where a variety of district level ARTI estimates were presented (bimodal distribution) [50], and much higher than the 0.1% ARTI estimate from Kassala State, Sudan (distribution not provided, ≥10mm threshold) [51]. Published estimates of the ARTI in Central African Republic do not account for striking digit preference and are likely, therefore, to overestimate the force of infection [52].

The extraordinary force of infection in South Africa’s Western Cape province is notable. HIV prevalence is lower than in KZN. Western Cape is more affluent. Potential explanations include cool winters leading to more indoor contact and Cape Town’s grossly unequal geography requiring the population to undertake long commutes on public transport to access employment and services [53]. Differences in circulating *Mtb* strains may also explain some of this difference, with Beijing strain comprising a greater proportion of cases in both Eastern and Western Cape than in other provinces of South Africa [54, 55].

Critiques of the various approaches to measuring *Mtb* transmission at the community level are presented elsewhere [1, 5]. An attempt to document infection incidence in adolescents using serial interferon gamma release assays (IGRA) is planned in the same community as that in which we undertook our TST survey. This follows an infection prevalence survey in adolescents completed in 2017–18 [56]. Interestingly, back calculating ARTI from age specific infection prevalence using data from the 2017–18 survey yields estimates similar to those we measured in primary school children. These estimates and discussion as to why an increase in ARTI with age was, apparently, not seen can be found in S1 Text. We await the results of the infection incidence survey with interest.

A major strength of this survey was that, by nesting it within a household surveillance programme, we could estimate how participating children differed from children that did not participate. Participation was somewhat higher in children living in more rural areas.

There are some limitations to this survey. The study did not include formal validation of TST readings. Nearly all of the tests were administered and read by a single clinician (SC). A second clinician (TAY) observed and replicated many of these readings. However, formally quantifying agreement between TST readings by undertaking blinded double reading of a random sample of tests would have been informative.

We did not test children for HIV or collect data on children’s HIV status. In the 2005–6 birth cohort, local HIV prevalence is estimated to be less than 5% (personal communication: Dr James Ndirangu, 2013). This assumes an antenatal HIV prevalence of approximately 40%, a vertical transmission rate at that time of approximately 15% and substantial early mortality. Therefore, in approximately one in twenty children in this study, the TST may have had reduced sensitivity [57]. This may have led to an underestimate of infection prevalence, particularly if HIV positive children had greater exposure to *Mtb* than other children.

It is theoretically possible that HIV negative children exposed to HIV *in utero* might be more likely to have anergic TSTs. In Malawi, young children with HIV positive mothers were more likely to have positive TSTs, likely reflecting greater *Mtb* exposure [58]. In the same survey, the sizes of non zero reactions were similar among children with HIV positive mothers and children with HIV negative mothers (as in our survey, these children were not tested for HIV) [58].

Of the 5690 children registered in the surveillance programme who were born in 2005–6, 281 (4.9%) had died by 25 June 2013. By verbal autopsy, the major causes of death in young children in this community were acute respiratory infections, HIV/AIDS and birth asphyxia [19]. If *Mtb* infected children had a higher mortality than uninfected children, infection prevalence might have been underestimated. It is not usual to account for differential survival when analysing tuberculin survey data.

The overall impact of HIV associated TST anergy and differential survival on our ARTI estimates is expected to be limited given these children represent a small proportion of the overall sample.

A further reason we may have underestimated *Mtb* transmission is that we used cross-sectional data. Fine noted, ‘Conventional cross-sectional analysis methods give an accurate estimate of incidence only if there is in fact no reversion, and if there are equal numbers of false negative and false positive tests’ [27]. Test reversions may mean that longitudinal studies tend to produce higher ARTI estimates than do cross sectional studies. Whilst previous descriptions of this phenomenon using IGRAs [59] might have been a consequence of readings around the threshold being unreliable [60], TST reversions have been documented to occur in longitudinal data, even applying stringent definitions of test conversion [27]. Additional data on the association between test reversions and subsequent TB incidence would be helpful in understanding to what extent reversions represent initial false positive tests versus exposure to *Mtb*. Regardless, our survey provides ARTI estimates that allow comparison with other similar cross sectional studies.

Our analysis assumes a stable incidence of *Mtb* infection. Longitudinal data on the force of infection in KZN don’t exist and we are not aware of data on changes in the force of infection over the period 2005–2013 from elsewhere in South Africa. The incidence of microbiologically confirmed pulmonary TB in South Africa is thought to have peaked in approximately 2008 [17]. There is some suggestion that the peak occurred later in KZN, although comparable data were only available in KZN from 2011 onward [17]. The incidence of diagnosed disease is determined by the force of infection, but also rates of progression from infection to disease and the capacity of the healthcare system. It seems likely that changes in TB notifications over this period were mainly driven by changes in rates of progression to disease, given this period saw the rapid roll out of antiretroviral therapy in the public sector [17, 20, 61]. If ARTI were rising or falling, our estimate would best reflect the force of infection midway between these children being born and being tested (i.e. approximately 2009).

Finally, whilst these data may be useful in estimating differences in force of infection over time or (with caution) between communities, it is likely that our headline ARTI estimates significantly understate the true force of infection in the community [62]. As discussed above, test reversions might lead to cross sectional surveys underestimating the ARTI. TST surveys enrolling children can underestimate transmission in communities where mixing patterns are age assortative. This is expected where children have less contact with adult men, a demographic disproportionately likely to have untreated pulmonary TB [41–44]. ARTI can also be underestimated because some individuals fail to convert their TST/IGRA despite heavy exposure [63–65]. Naively applying ARTI estimates of the kind we present here can lead control strategies to underemphasise the importance of recent transmission and, particularly in high burden setting, to place undue emphasis on interventions seeking to prevent reactivation of remote infection [62].

A comprehensive discussion of potential approaches to limiting *Mtb* transmission in this community is beyond the scope of this manuscript. There are reasons to believe that further improvements in case finding and treatment, whilst clearly important, may not be sufficient [3, 66]. Whilst expanding antiretroviral coverage will undoubtedly reduce TB incidence and mortality, the impact on transmission is less predictable [1, 2, 67, 68]. Potential alternative approaches to reducing transmission at a community level include making TB services more accessible to men as, in South Africa, more than half of *Mtb* infections in men, women and children are a result of contact with adult men with prevalent pulmonary TB [41, 43, 44, 69]; interventions targeted at individuals who have previously had TB, a group who suffer a disproportionate burden of TB disease [70]; improvements in natural ventilation in indoor public spaces [71, 72]; improving TB infection control in healthcare facilities [73]; and action to improve socioeconomic conditions [74, 75].

## Conclusion

The force of infection in KZN is higher than that seen in most of the world, but lower than that observed in the Western Cape of South Africa. Mixture analysis proved unstable, likely a result of our modest sample size, but the two possible results yielded similar estimates of the ARTI. As in other high burden settings, the high CIR and modest association between household TB contact and TST positivity is in keeping with children commonly acquiring *Mtb* outside the home. Additional action is urgently needed to interrupt *Mtb* transmission in this and similar communities.

## Supporting information

S1 Text Supplementary material. 1. Approaches to defining TST thresholds, 2. A critique of the household asset score, 3. Summary level data from the 1974 and 1979 TST surveys, 4. Estimating Annual Risk of Tuberculous Infection in adolescents from infection prevalence data, Fig A. The distribution of household asset scores in 2003, Fig B. The distribution of household asset scores in 2009, Fig C. The distribution of household asset scores in 2013, Table A. Cross-sectional TST surveys performed by the SA Medical Research Council between 1974 and 1979 in KwaZulu-Natal, South Africa, Table B. TST results in rural children in KwaZulu-Natal, South Africa, surveyed in 1974 by the SA Medical Research Council, Table C. TST results in rural children in KwaZulu-Natal, South Africa, surveyed in 1974 by the SA Medical Research Council, Table D. TST results in rural children in KwaZulu-Natal, South Africa, surveyed in 1979 by the SA Medical Research Council, Table E. TST results in rural children in KwaZulu-Natal, South Africa, surveyed in 1979 by the SA Medical Research Council, Table F. ARTI calculated from infection prevalence data in adolescents.
(DOCX)

S1 Checklist STROBE checklist. (DOCX)

S2 Checklist PLOS inclusivity in global research questionnaire. (DOCX)
