# Supplementary material for: Tuberculin skin test surveys and the Annual Risk of Tuberculous Infection in school children in Northern KwaZulu-Natal
Source: PLOS Glob Public Health. 2024 Jun 18;4(6):e0003263. doi: 10.1371/journal.pgph.0003263 (PMC11185501; doi:10.1371/journal.pgph.0003263)
Supplement: S1 Text — 1. Approaches to defining TST thresholds, 2. A critique of the household asset score, 3. Summary level data from the 1974 and 1979 TST surveys, 4. Estimating Annual Risk of Tuberculous Infection in adolescents from infection prevalence data, Fig A. The distribution of household asset scores in 2003, Fig B. The distribution of household asset scores in 2009, Fig C. The distribution of household asset scores in 2013, Table A. Cross-sectional TST surveys performed by the SA Medical Research Council between 1974 and 1979 in KwaZulu-Natal, South Africa, Table B. TST results in rural children in KwaZulu-Natal, South Africa, surveyed in 1974 by the SA Medical Research Council, Table C. TST results in rural children in KwaZulu-Natal, South Africa, surveyed in 1974 by the SA Medical Research Council, Table D. TST results in rural children in KwaZulu-Natal, South Africa, surveyed in 1979 by the SA Medical Research Council, Table E. TST results in rural children in KwaZulu-Natal, South Africa, surveyed in 1979 by the SA Medical Research Council, Table F. ARTI calculated from infection prevalence data in adolescents. (DOCX) [file pgph.0003263.s001.docx]

**Supplement**

**Manuscript title:** Annual Risk of Tuberculous Infection in Northern KwaZulu-Natal

**Authors:** Tom A. Yates, Siphiwe Cebekhulu, Mumsy Mthethwa, Marie-Louise Newell, Ibrahim Abubakar, Frank Tanser

**Table of contents**

Approaches to defining TST thresholds 2

The household asset score – assets used, and a critique 3

Summary level data from the 1974 and 1979 TST surveys 5

Estimating Annual Risk of Tuberculous Infection in adolescents from infection prevalence data 7

**1. Approaches to defining TST thresholds**

Several standard approaches to defining TST thresholds are described in the literature. These methods are described below. A more detailed account of these approaches, including their theoretical underpinning (1); discussion of the appropriate use of TST and Interferon Gamma Release Assays in clinical decision making (2,3); plus discussion of mixture analysis (4–6) and the Rust and Thomas approach (7–9), two alternatives to these standard approaches, can be found elsewhere. Some investigators apply these methods to smoothed data, which can be helpful where distributions are noisy or where there is clear digit preference.

Perhaps the most commonly applied TST threshold is to consider reactions of ≥10mm to be positive. Given the frequency and the distributions of both reactions to *Mycobacterium tuberculosis* (*MTb*) and non specific reactions appear to be setting dependent, the use of set thresholds of this kind may discriminate poorly between reactions as a result of *MTb* infection and non specific TST reactions. For example, in populations with no exposure to non-tuberculous mycobacteria and no history of recent BCG vaccination, all positive TST reactions are likely to represent exposure to *MTb*.

A fixed threshold of ≥15mm is also commonly also applied. Whilst it is unlikely that this threshold will ever best discriminate between reactions to *MTb* and non-specific reactions, this threshold offers the advantage of greater specificity.

An alternative approach assumes that all reactions ≥14mm are a result of *MTb* infection (a strong assumption, not fully supported by data). Infection prevalence is then obtained by multiplying this proportion by 1.22. This figure is derived from the sensitivity of the ≥14mm threshold (82%) in data from a setting with little cross reactivity (1/0.82 = 1.22).

There are also ‘mirror’ methods. These methods assume that all reactions greater than or equal to the mode represent *MTb* infection, an assumption that is more reasonable with higher modes. They also assume that TST reactions resulting from *MTb* exposure are approximately normally distributed, i.e. that the number of these reactions above the mode approximates the number below the mode. Under these assumptions, infection prevalence can be estimated by summing TST reactions > and ≥ the mode. The standard mirror method uses the modal value from the data – note, this is not always clear, particularly in smaller datasets. The ‘fixed’ mirror method applies the same approach with a ‘mode’ of 17mm, a value that is commonly observed in surveys. Mirror methods throw away a lot of data and infection prevalence estimates derived from mirror methods are highly sensitive to the modal value chosen.

**2. The household asset score – assets used, and a critique**

The following assets were included in the household asset score: bed net, bed, bicycle, block maker, car, car battery, cattle, electric hotplate, electric kettle, fridge, gas cooker, Kombi/lorry/tractor, kitchen sink, motorcycle, other livestock, Primus stove, radio, sofa, sewing machine, table and chairs, telephone, cell phone, TV, video, wheelbarrow, plus the fuel used for cooking and the sanitation facilities available with the latter two assets converted into sets of dummy binary variables.

In assessing the performance of household asset scores in discriminating between individuals of higher versus lower socio-economic position, it can be useful to plot the distribution of household asset scores across the population. (10) The figures below show these distributions when our asset score is applied in the same community using data from 2003, 2009 and 2013. It can be observed that the distribution of household asset scores has shifted to the right over time, presumably a consequence of more widespread ownership of fridges, cell phones, and the other assets used to construct the score. To address this truncation and ensure the asset score can continue to discriminate between households, it may be necessary to include additional data on assets that are not yet widely owned.

**Fig A. The distribution of household asset scores in 2003**

**Fig B. The distribution of household asset scores in 2009**

**Fig C. The distribution of household asset scores in 2013.**

**3. Summary level data from the 1974 and 1979 TST surveys**

The following data were kindly provided by Prof Bernard Fourie and relate to TST surveys conducted in black children in KwaZulu-Natal (KZN) by the South African Medical Research Council (11,12). As described in Methods, children with no TST reaction were included in the 0-1mm bracket, with the 0-1mm category including reactions of between zero and 1.9mm, the next category including reactions of between 2.0mm and 3.9mm, etc.

**Table A. Cross-sectional TST surveys performed by the SA Medical Research Council between 1974 and 1979 in KwaZulu-Natal, South Africa**

| **Location** | **Survey year** | **Age cohort (yrs)** | **Tests performed (N)** | |
| --- | --- | --- | --- | --- |
|  |  |  | **All** | **No BCG scar** |
| Urban KZN - Durban | 1976  1977 | 5-9  5-9 | 1507  1854 | 1284  1741 |
| Urban KZN - Pietermaritzburg | 1978 | 5-9  10-14  15-19 | 183  563  586 | 147  328  405 |
| Rural KZN | 1974  1979 | 0-4  5-9  10-14  15-19  0-4  5-9  10-14  15-19  20-29 | 532  523  500  208  361  453  370  220  179 | 435  389  291  133  301  352  266  132  138 |

**Table B. TST results in rural children in KwaZulu-Natal, South Africa, surveyed in 1974 by the SA Medical Research Council**

| **Age (yrs)** | **Mantoux test result (mm) in subjects without BCG scars** | | | | | | | | | | | | | | | | **Total** |
| --- | --- | --- | --- | --- | --- | --- | --- | --- | --- | --- | --- | --- | --- | --- | --- | --- | --- |
|  | **0-1** | **2-3** | **4-5** | **6-7** | **8-9** | **10-11** | **12-13** | **14-15** | **16-17** | **18-19** | **20-21** | **22-23** | **24-25** | **26-27** | **28-29** | **>=30** |  |
| 0-4 | 204 | 167 | 37 | 6 | 2 | 3 | 1 | 2 | 7 | 3 | 1 | 2 | - | - | - | - | 435 |
| 5-9 | 151 | 144 | 38 | 8 | 8 | 5 | 4 | 10 | 5 | 7 | 6 | - | 1 | - | - | 2 | 389 |
| 10-14 | 78 | 85 | 27 | 10 | 21 | 16 | 14 | 11 | 12 | 9 | 3 | 2 | 2 | 1 | - | - | 291 |
| 15-19 | 31 | 17 | 15 | 13 | 6 | 10 | 12 | 11 | 9 | 6 | 1 | 2 | - | - | - | - | 133 |

**Table C. TST results in rural children in KwaZulu-Natal, South Africa, surveyed in 1974 by the SA Medical Research Council**

| **Age (yrs)** | **Mantoux test result (mm) in subjects with/without BCG scars** | | | | | | | | | | | | | | | | **Total** |
| --- | --- | --- | --- | --- | --- | --- | --- | --- | --- | --- | --- | --- | --- | --- | --- | --- | --- |
|  | **0-1** | **2-3** | **4-5** | **6-7** | **8-9** | **10-11** | **12-13** | **14-15** | **16-17** | **18-19** | **20-21** | **22-23** | **24-25** | **26-27** | **28-29** | **>=30** |  |
| 0-4 | 274 | 179 | 45 | 7 | 3 | 3 | 2 | 4 | 8 | 4 | 1 | 2 | - | - | - | - | 532 |
| 5-9 | 219 | 164 | 51 | 13 | 10 | 11 | 7 | 16 | 9 | 9 | 8 | 2 | 1 | 1 | - | 2 | 523 |
| 10-14 | 119 | 109 | 42 | 22 | 37 | 36 | 30 | 37 | 30 | 20 | 11 | 4 | 2 | 1 | - | - | 500 |
| 15-19 | 41 | 20 | 17 | 19 | 12 | 19 | 24 | 16 | 20 | 13 | 3 | 2 | - | 1 | - | 1 | 208 |

**Table D. TST results in rural children in KwaZulu-Natal, South Africa, surveyed in 1979 by the SA Medical Research Council**

| **Age (yrs)** | **Mantoux test result (mm) in subjects without BCG scars** | | | | | | | | | | | | | | | | **Total** |
| --- | --- | --- | --- | --- | --- | --- | --- | --- | --- | --- | --- | --- | --- | --- | --- | --- | --- |
|  | **0-1** | **2-3** | **4-5** | **6-7** | **8-9** | **10-11** | **12-13** | **14-15** | **16-17** | **18-19** | **20-21** | **22-23** | **24-25** | **26-27** | **28-29** | **>=30** |  |
| 0-4 | 268 | 1 | 7 | 7 | 6 | 4 | - | - | 1 | 3 | 2 | 1 | - | - | - | 1 | 301 |
| 5-9 | 275 | 1 | 12 | 15 | 9 | 5 | 6 | 2 | 3 | 3 | 3 | 4 | 5 | 7 | 2 | - | 352 |
| 10-14 | 176 | 4 | 9 | 19 | 12 | 10 | 6 | 6 | 3 | 1 | 6 | 2 | 2 | 3 | 1 | 6 | 266 |
| 15-19 | 64 |  | 5 | 10 | 10 | 10 | 5 | 5 | 5 | 4 | 5 | 1 | 2 | 3 | 2 | 1 | 132 |

**Table E. TST results in rural children in KwaZulu-Natal, South Africa, surveyed in 1979 by the SA Medical Research Council**

| **Age (yrs)** | **Mantoux test result (mm) in subjects with/without BCG scars** | | | | | | | | | | | | | | | | **Total** |
| --- | --- | --- | --- | --- | --- | --- | --- | --- | --- | --- | --- | --- | --- | --- | --- | --- | --- |
|  | **0-1** | **2-3** | **4-5** | **6-7** | **8-9** | **10-11** | **12-13** | **14-15** | **16-17** | **18-19** | **20-21** | **22-23** | **24-25** | **26-27** | **28-29** | **>=30** |  |
| 0-4 | 309 | 2 | 10 | 10 | 12 | 4 | 1 | 3 | 3 | 3 | 2 | 1 | - | - | - | 1 | 361 |
| 5-9 | 305 | 1 | 16 | 28 | 15 | 12 | 15 | 6 | 8 | 14 | 8 | 6 | 7 | 8 | 4 | - | 453 |
| 10-14 | 202 | 5 | 14 | 26 | 18 | 16 | 15 | 13 | 5 | 9 | 12 | 6 | 8 | 7 | 4 | 10 | 370 |
| 15-19 | 76 | 1 | 9 | 18 | 19 | 16 | 11 | 16 | 10 | 13 | 11 | 4 | 5 | 5 | 4 | 2 | 220 |

**4. Estimating Annual Risk of Tuberculous Infection in adolescents using infection prevalence data**

A cross sectional infection prevalence survey enrolling adolescents was undertaken in the same community in 2017-18. (13) The survey used the QuantiFERON-TB Gold-plus assay and applied the threshold recommended by the manufacturer (≥0.35 IU/ml). Annual Risk of Tuberculous Infection (ARTI) was calculated using the approach described in Methods. These calculations used infection prevalence data from the 2017-18 survey disaggregated by age and make assumptions about the mean age at testing within each age bracket (see table).

**Table F. ARTI calculated from infection prevalence data in adolescents**

| Age in years | Infection prevalence (%) | Mean age at testing (assumed) | ARTI (%) |
| --- | --- | --- | --- |
| 10-11 | 49/237 (20.7) | 11.0 | 2.1 |
| 12-14 | 62/349 (17.8) | 13.5 | 1.4 |
| 15-17 | 71/297 (23.9) | 16.5 | 1.6 |
| ≥18 | 67/211 (31.8) | 20.0 | 1.9 |

As discussed in the main manuscript, strong assumptions are needed to back calculate ARTI from infection prevalence – in particular, that the force of infection does not vary by age and that it has been constant over the period that these children have been alive. These assumptions are likely to be less reasonable in older individuals.

With these limitations in mind, it is striking that the ARTI estimates in these older individuals were no higher than those estimated among 6-8 year olds in my survey. It is generally accepted that ARTI increases through adolescence - this literature is reviewed elsewhere (14). Even averaging across these individual’s lifetimes, one might expect ARTI to be higher in adolescents than in young children.

One potential explanation is that ARTI fell between 2013 and 2017-18. There is no good data to support this idea. As discussed in the main manuscript, falls in TB notifications over this period were likely a result of expanded provision of antiretroviral therapy to people living with HIV. (15)

Perhaps more likely, ARTI does increase with age but current tools may not capture this in settings where the force of infection is high. First, individuals most at risk of infection as a result, for example, of their contact network would be infected first then also be most at risk of reinfection. Current tests for *MTb* infection do not allow us to observe reinfections. Second, a proportion of individuals do not mount TST or IGRA responses even following heavy exposure and, over time, these individuals make up an increasing proportion of those ‘uninfected’. (14) Data on incident *MTb* infections in adolescents in this community are expected and will be informative.

1. Rieder HL. Epidemiologic Basis of Tuberculosis Control. 1st ed. Paris: International Union Against Tuberculosis and Lung Disease; 1999. 164 p.

2. Pai M, Denkinger CM, Kik S V., Rangaka MX, Zwerling A, Oxlade O, et al. Gamma interferon release assays for detection of Mycobacterium tuberculosis infection. Clin Microbiol Rev. 2014;27(1):3–20.

3. Gupta RK, Calderwood CJ, Yavlinsky A, Krutikov M, Quartagno M, Aichelburg MC, et al. Discovery and validation of a personalized risk predictor for incident tuberculosis in low transmission settings. Nat Med. 2020;26(12):1941–9.

4. Neuenschwander BE, Zwahlen M, Kim SJ, Lee EG, Rieder HL. Determination of the prevalence of infection with Mycobacterium tuberculosis among persons vaccinated against Bacillus Calmette-Guérin in South Korea. American Journal of Epidemiology. 2002;155(7):654–63.

5. Neuenschwander B. Bayesian Mixture Analysis for Tuberculin Induration Data. 2003;(July).

6. Davies GR, Fine PE, Vynnycky E. Mixture analysis of tuberculin survey data from northern Malawi and critique of the method. International Journal of Tuberculosis and Lung Disease. 2006;10(9):1023–9.

7. Rust P, Thomas J. A method for estimating the prevalence of tuberculosis infection. Am J Epidemiol. 1975;101(4):311–22.

8. Rust P. Standard errors in mixture studies of tuberculous infection prevalence. International Journal of Tuberculosis and Lung Disease. 2003;7(2):194–7.

9. Khan PY, Glynn JR, Mzembe T, Mulawa D, Chiumya R, Crampin AC, et al. Challenges in the Estimation of the Annual Risk of Mycobacterium tuberculosis Infection in Children Aged Less Than 5 Years. American journal of epidemiology. 2017;186(8):1015–22.

10. Vyas S, Kumaranayake L. Constructing socio-economic status indices: How to use principal components analysis. Health Policy and Planning. 2006;21(6):459–68.

11. Fourie P. Patterns of tuberculin hypersensitivity in South Africa. Tubercle. 1983;64:167–79.

12. Fourie PB. The prevalence and annual rate of tuberculous infection in South Africa. Tubercle. 1983;64(3):181–92.

13. Mzembe T, Lessells R, Karat AS, Randera-Rees S, Edwards A, Khan P, et al. Prevalence and Risk Factors for Mycobacterium tuberculosis Infection among Adolescents in Rural South Africa. Open Forum Infectious Diseases. 2021;8(1):1–8.

14. Dowdy DW, Behr MA. Personal View Are we underestimating the annual risk of infection with Mycobacterium tuberculosis in high-burden settings? The Lancet Infectious Diseases. 2022;3099(22):1–8.

15. Nanoo A, Izu A, Ismail NA, Ihekweazu C, Abubakar I, Mametja D, et al. Nationwide and regional incidence of microbiologically confirmed pulmonary tuberculosis in South Africa, 2004-12: A time series analysis. The Lancet Infectious Diseases. 2015;15(9):1066–76.
